# 11554 Getting the Grant: Assessment of a Monthly Grant Writing Group for Junior Investigators

**DOI:** 10.1017/cts.2021.557

**Published:** 2021-03-30

**Authors:** Lauren Aleksunes, Yasheca Ebanks, Suzie Chen

**Affiliations:** Rutgers University

## Abstract

ABSTRACT IMPACT: NJ ACTS provides mentored coaching in NIH grant writing for early stage investigators. OBJECTIVES/GOALS: Launching an independent academic careers requires the ability to effectively communicate the purpose and impact of biomedical research in order to obtain extramural funding. We sought to develop and evaluate an interactive grant writing group of junior faculty and senior postdoctoral fellows mentored by trained coaches. METHODS/STUDY POPULATION: Participants meet monthly for 1 hour to peer review Specific Aims pages for grant applications to NIH and private foundations. Sessions are moderated by two senior faculty trained as coaches by the National Research Mentoring Network. Participant grant submission and review of the program are collected annually. RESULTS/ANTICIPATED RESULTS: From 2019-2020, 15 faculty and 2 postdoctoral fellows participated in the grant writing group with an average of 5.7 participants each month. Over the year, participants submitted 53 grant applications (68% submitted to NIH with the majority being R21 or R01 grant mechanisms). Half of grants submitted were discussed during peer review sessions. Of the grants reviewed, 42% were funded or near/below the funding payline. Using a 5-point Likert scale, participants highly rated the quality of coaching (mean/SD: 4.9 ±0.2), time discussing their research (mean/SD: 4.5 ±0.8), and the input from other participants (mean/SD: 4.5 ±0.5). DISCUSSION/SIGNIFICANCE OF FINDINGS: In conclusion, a monthly meeting of junior investigators hosted by two grant writing coaches is an effective means to receive peer review of grant application aims and support submissions for extramural funding.

